# P02.158. Tai Chi community program is effective in reducing elderly fall-related hospital utilization: a prospective observational study

**DOI:** 10.1186/1472-6882-12-S1-P214

**Published:** 2012-06-12

**Authors:** E Ziea

**Affiliations:** 1 Chinese Medicine Department, Hospital Authority of Hong Kong, Hong Kong, Hong Kong

## Purpose

Overseas studies and reviews showed that about 30% of elderly suffer from falls every year and approximately one fall in ten resulted in fractures. Fall is a major cause of hospitalization in Hong Kong, ranking the fifth in terms of total bed days of all public acute hospitals. Tai Chi (TC) is a Chinese martial art form for centuries and seems to be an effective intervention to prevent falls through improving functional balance and physical response.

## Methods

A prospective observational study of 3,222 community-dwelling elderly aged 60 or above was conducted to assess the effect of TC in reducing injurious falls incidence. Participants were community-dwelling elderly aged 60 or above with “Timed Up-and-Go” test (TUGT) less than or equal to 20 seconds. An hour simplified TC session led by recognized TC coaches was performed twice a week in a period of four weeks. Participants were encouraged to practice TC after training. Injurious fall incidents were retrieved and compared from a medical records database 12 months before and after TC training between 2 strata: continue (n=1,269) vs cease (n=1,360) TC in 12 months post training.

## Results

The injurious fall rate dropped from 2.21% (pre) to 1.34% (post) in the continuing TC group while the fall rate increased from 2.06% (pre) to 3.46% (post) in the non-continuing TC group. The percentage of hospitalization due to fall-related injuries was 0.39% and 1.03% in the continuing and non-continuing TC groups, respectively.

## Conclusion

Study results demonstrate that TC has a potential preventive effect on injurious falls and hospital utilization.

